# Evaluation of Intracellular Gene Transfers from Plastome to Nuclear Genome across Progressively Improved Assemblies for *Arabidopsis thaliana* and *Oryza sativa*

**DOI:** 10.3390/genes13091620

**Published:** 2022-09-09

**Authors:** Haoqi Wang, Xuezhu Liao, Luke R. Tembrock, Zuoren Yang, Zhiqiang Wu

**Affiliations:** 1 Zhengzhou Research Base, State Key Laboratory of Cotton Biology, Zhengzhou University, Zhengzhou 450001, China; 2 Shenzhen Branch, Guangdong Laboratory for Lingnan Modern Agriculture, Genome Analysis Laboratory of the Ministry of Agriculture, Agricultural Genomics Institute at Shenzhen, Shenzhen 518120, China; 3 Kunpeng Institute of Modern Agriculture at Foshan, Foshan 528200, China; 4 Department of Agricultural Biology, Colorado State University, Fort Collins, CO 80523, USA; 5 State Key Laboratory of Cotton Biology, Institute of Cotton Research, Chinese Academy of Agricultural Sciences, Anyang 455000, China

**Keywords:** intracellular gene transfer, plastid, NUPTs (nuclear plastid DNAs), genome evolution, genome assembly index

## Abstract

DNA originating from organellar genomes are regularly discovered in nuclear sequences during genome assembly. Nevertheless, such insertions are sometimes omitted during the process of nuclear genome assembly because the inserted DNA is assigned to organellar genomes, leading to a systematic underestimation of their frequency. With the rapid development of high-throughput sequencing technology, more inserted fragments from organelle genomes can now be detected. Therefore, it is necessary to be aware of the insertion events from organellar genomes during nuclear genome assembly to properly attribute the impact and rate of such insertions in the evolution of nuclear genomes. Here, we investigated the impact of intracellular gene transfer (IGT) from the plastome to the nuclear genome using genome assemblies that were refined through time with technological improvements from two model species, *Arabidopsis thaliana* and *Oryza sativa*. We found that IGT from the plastome to the nuclear genome is a dynamic and ongoing process in both *A. thaliana* and *O. sativa*, and mostly occurred recently, as the majority of transferred sequences showed over 95% sequence similarity with plastome sequences of origin. Differences in the plastome-to-nuclear genome IGT between *A. thaliana* and *O. sativa* varied among the different assembly versions and were associated with the quality of the nuclear genome assembly. IGTs from the plastome to nuclear genome occurred more frequently in intergenic regions, which were often associated with transposable elements (TEs). This study provides new insights into intracellular genome evolution and nuclear genome assembly by characterizing and comparing IGT from the plastome into the nuclear genome for two model plant species.

## 1. Introduction

As the first plant reference genome, the genome sequence of *A. thaliana* was published in December 2000 [1], and heralded the beginning of the plant genome sequencing era. Since then, the sequencing of plant genomes has made significant progress over the last 20 years. With the continuous development of sequencing technologies and the decreasing cost of such technologies, the quality of genome assembly has been significantly improved [2], even achieving T2T levels for some species [3,4,5].

The continuous development and progress of sequencing technology has led to the completion of genomes from a diversity of plant species. However, the early whole genome sequencing of *A. thaliana* [1], *O. sativa* [6], and other species relied on first-generation DNA sequencing technology such as the dideoxy chain termination method proposed by Sanger, et al. [7]. The read length of Sanger sequencing technology cannot exceed 1000 bp, nor can different amplicons be processed simultaneously, making it a low throughput and high-cost method unsuitable for large-scale sequencing projects. Next-generation sequencing (NGS) technologies expanded on Sanger sequencing by developing methods for reading many amplicons simultaneously, but because these methods still relied on sequencing by synthesis, the read lengths generally did not exceed those of Sanger sequencing. The high throughput and low cost of NGS technology, along with improvements in bioinformatic algorithms needed for assembling genomes from such data, resulted in the large expansion in the number of plant genomes being published [8,9,10]. Subsequently, the advent of third-generation sequencing technologies has enabled the generation of 10 Kb or longer reads, thus greatly improving genome assembly continuity, especially in genomic regions with abundant repeats [11]. Based on the most recent sequencing and assembly technologies, many researchers have worked to improve the quality and completeness of genomes from model species, such as *A. thaliana* and *O. sativa* in an effort to describe all genomic features to the nucleotide level. For example, the genome of *A. thaliana* has been continuously updated since it was first published in 2000, ranging from NGS data with numerous Ns [1] to third-generation sequencing data such as ONT and PacBio along with Hi-C sequencing with some gaps [12], to the current gap-free version using ONT and PacBio HiFi long-read sequencing [4]. In addition, many different evaluation indices have been applied to measure the quality and continuity of genome assembly including BUSCO (Benchmarking Universal Single-Copy Orthologs), CEGMA (Core Eukaryotic Genes Mapping Approach), and LAI (LTR Assembly Index) [13,14,15]. However, what the effect of genome assembly is on intracellular gene transfer (IGT) detection as a function of sequencing technology and assembly algorithm has not been thoroughly assessed.

Horizontal gene transfer (HGT, also sometimes referred to as lateral gene transfer, LGT) refers to the exchange of genetic material between individuals from divergent lineages [16,17]. In contrast to vertical gene transfer (VGT) from parent to offspring, HGT is the transfer of genetic material between individuals isolated by reproductive barriers. The presence of HGT segments in a genome can complicate inferences of relatedness by obscuring signals of divergence at the sequence level but, if analyzed at the structural level may, if properly identified, provide a useful marker for delimiting lineages with a distinct insertion [18,19].

IGT is similar to HGT but involves the transfer of genetic material between cellular compartments. It is generally defined as the mutual transfer of DNA between organelle genomes (mitogenome and plastome) and the nuclear genome within the cell, and is sometimes referred to as endosymbiotic gene transfer (EGT) [20,21]. DNA transfer in plants has been extensively documented, not only from organelles to the nucleus, but also between organelles [22,23,24]. There are three possible types of sequence transfer, that is, the transfer of nuclear sequences into the mitochondrial or plastid genome, the transfer of mitochondrial sequences into the nucleus or plastid genome, and the transfer of plastid sequences into the nuclear or mitochondrial genome [20]. Among these types of transfers, the most common IGTs are from the organellar genomes to the nuclear genome, producing nuclear mitochondrial DNAs (NUMTs) and nuclear plastid DNAs (NUPTs). For instance, a large number of NUPTs have been found in the *A. thaliana* and *O. sativa* genomes [25], as well as a 52-Kb NUMT in the *Triticum* lineage [26]. The structure and gene content of the plastome is generally conserved among different land plant species [27], making detection of recently inserted NUPTs tractable provided sequencing reads span enough flanking nuclear DNA. Compared to mitogenomes, plastomes are not only conserved [28], but also lack an efficient DNA uptake apparatus [29]. Due to the above characteristics of plastomes, it is thought that nuclear-to-plastid gene transfers occur very infrequently, if at all [21,30]. In fact, of the six possible directions of IGT between the three genomes of plant cells, the only one that has not been identified is nuclear-to-plastid transfer [17,31]. Most work on IGTs from the plastome to nucleus has focused on how patterns of transfer differ between species or genes and gene regions [32,33,34], with little to no work examining how different sequencing technologies and assembly methods might affect the inference of IGT.

The rapid development of next-generation sequencing technologies has made it possible to study IGT events in greater detail and with improved accuracy. The assembly of plant genomes from frequently studied species such as *A. thaliana* and *O. sativa* is now considered to be at the chromosome level and pan-genomic. Such genomic datasets spanning multiple sequencing technologies allows researchers to study the effects of IGTs on genome assembly and the number of IGTs inferred as a function of sequencing technology. Here, from the perspective of comparatively assessing whether the detection of IGT events can be reflective of the quality of nuclear genome assembly, we analyzed plastome transfers to the nuclear genome between different sequencing and assembly versions of *A. thaliana* and *O. sativa*. Ultimately, such efforts will allow researchers to better characterize how such transfers affect genomic evolution.

## 2. Methods

### 2.1. Data Sampling

#### 2.1.1. Acquisition of Genomic Data

Nuclear genome sequences, along with gene annotations of different assembled versions of two model species (*A. thaliana* and *O. sativa*), were downloaded with data sources and basic information listed in Table 1 and Table 2. The four assembled versions of *O. sativa* are 9311 (assembled in 2002, using whole-genome shotgun illumina sequencing, [6]), SH498 (assembled in 2017, using PacBio sequencing, [35]), and MH63 and ZS97 (assembled in 2021, using the PacBio Sequel II sequencing platform, [5]). The four versions of *A. thaliana* are Phy13 (assembled in 2013, Phytozome13/*Athaliana*_167_TAIR10), T10 (assembled in 2014, NCBI/GCA_000001735.2_TAIR10.1), GWH (assembled in 2021, using Oxford Nanopore Technology and PacBio along with Hi-C sequencing, [12]), and Col (assembled in 2021, using ONT and PacBio HiFi long-read sequencing, [4]). Plastome sequence accession number were: *A. thaliana*, NC_000932.1 and *O. sativa*, NC_008155.1.

#### 2.1.2. Quality Assessment

BUSCO v5.2.2 [15] and QUAST v5.0.2 [36] were used to evaluate the assembly quality of the genomic data (Supplementary Figures S1 and S2, Table 1 and Table 2), and MUMmer v3.23 [37] was used to assess the collinearity of data between different versions. Pairwise comparison was performed on the above genomic data (see Supplementary Figures S3 and S4 for details). SeqKit v2.0.0 [38] and Bioawk v1.0 (https://github.com/lh3/bioawk, accessed on 25 August 2015) were used to calculate the number of Ns and chromosome length of genomic data for each version (Supplementary Table S1).

### 2.2. Analysis of IGT

#### Sequence Alignment

BLASTn v2.5.0+ [39] was used to align plastome sequences and nuclear genome sequences of each version, respectively. We set an e-value cutoff of 1 × 10^−6^.

### 2.3. Plastomes Transferred to Nuclear Chromosomes

Comparison of plastome sequences transferred to nuclear chromosomes between different versions of nuclear genomic data was conducted by first extracting the results based on the annotation information of the published sequences, and binning sequences into two categories (identity within 80–95% and identity ≥ 95%) to represent sequence transfers that are more ancient (accumulation of more mutations) and those that occurred more recently (accumulation of fewer mutations). Here, we are mainly concerned with the exact alignments with a similarity greater than or equal to 95%. We also set four size intervals (less than 100 bp, 100–500 bp, 500–1000 bp, and more than 1000 bp (1000 bp+)) to quantify how different lengths of transferred fragments contribute to overall IGT. Circos v0.69-8 [40] and LINKVIEW v1.0 (https://github.com/YangJianshun/LINKVIEW, accessed on 28 December 2019) were used to map the distribution of the transferred fragments on the nuclear genome. We displayed all aligned fragments with length ≥ 100 bp and identity ≥ 80% in Circos plots and highlighted alignments with identity ≥ 95% to characterize the distribution and number of plastome transferred fragments on nuclear chromosomes in different assembly versions. EDTA v1.9.4 [41] was used to annotate the repeated sequences of the nuclear genome and the TE content in the corresponding size intervals mentioned above.

### 2.4. Plastomes Transferred to Nuclear Genomic Regions

Difference analysis of plastome transfer to nuclear genic region between different versions of nuclear genomic data was conducted by extracting the bed file which records the location information of exons, introns, and intergenic regions of each version of the data according to the annotation file information. Then, the length and number of the transferred fragments in the different genomic regions and the length and number of TEs in the corresponding categories were quantified to assess the differences between different versions with the methods described above.

### 2.5. Characteristics of the Transferred Fragments

Characteristics analyses of the transferred fragments and the flanking sequences was performed using BEDTools v2.30.0 [42] and SeqKit v2.0.0 [38] to quantify the GC content of the plastome transfer segments and its two flanking sequences (including 100 bp, 500 bp, 1000 bp, and 2000 bp on either side of the transferred segment), as well as the length and number of TEs in the corresponding categories, including any internal TEs in the transferred fragments and the TEs of the two flanking sequences (any TE external to the transferred fragments at the above mentioned distances).

## 3. Results

### 3.1. Patterns in IGT from Plastome to Nuclear Genome in A. thaliana and O. sativa

**Figure 1:**
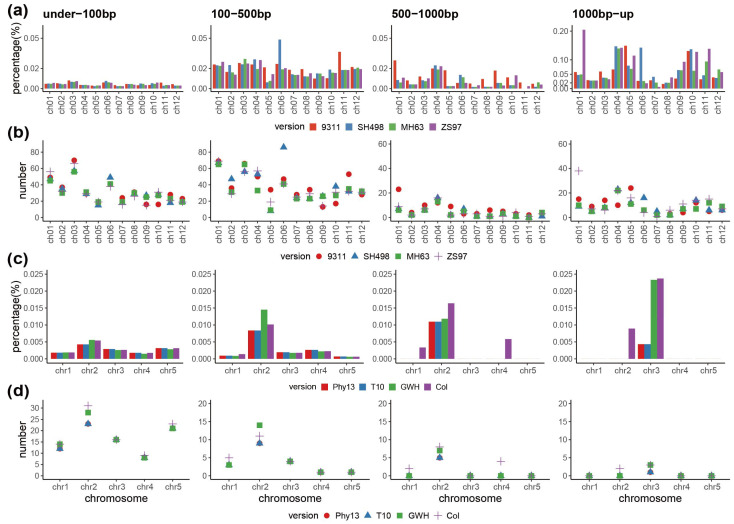
The length ratio and number of transferred plastome sequences in nuclear chromosomes in *O. sativa* and *A. thaliana* with 95% and higher identity. (**a**) Length proportion of plastome transfers to nuclear chromosomes in *O. sativa* across four different size categories. (**b**) The number of plastome sequences transferred to nuclear chromosomes in *O. sativa* across four different size categories. (**c**) Length proportion of plastome transfers to nuclear chromosomes in *A. thaliana* across four different size categories. (**d**) The number of plastome sequences transferred to nuclear chromosomes in *A. thaliana* across four different size categories. The bar charts represent the relative length of all transferred fragments to the total length of each nuclear chromosome as a percent, assembly versions are ordered along the x axis from oldest to newest.

To verify whether the IGT from plastome to nuclear chromosome differed between a monocotyledonous and dicotyledonous species, we calculated the relative length and number of different versions of plastome sequences transferred to nuclear chromosomes in *O. sativa* and *A. thaliana*, respectively. The results indicated that when identity is 95% or higher, the length and number of transferred fragments are generally higher than the bin with an identity between 80–95% in *A. thaliana* and *O. sativa* (Figure 1, Supplementary Figure S5), suggesting that most plastome-to-nuclear genome IGT events have occurred recently or that older sequences have been purged [18,43].

Results for the relative length and number of transferred fragments in *O. sativa* (Figure 1a,b) revealed that transferred sequences of 1000 bp+ made up a greater proportion of the nuclear genome than all shorter length categories, but that the two shortest categories were more frequently transferred than the longer categories. Differences between the *O. sativa* assembly versions were particularly apparent on chromosome 6, where in the 100–500 bp category the number of transfers varied by more than 40 between SH498 and the other versions, and the proportion of the nuclear genome made up of transfer sequences in the 1000 bp+ category was well over 0.10% in SH498 and less than 0.05% in all other versions (Figure 1a,b). As with *O. sativa*, in *A. thaliana*, the shorter transferred sequences occurred in higher numbers, but the longer sequences made up a greater proportion of the nuclear genome (Figure 1c,d). Especially in the 1000 bp+ category, the proportion of the nuclear genome made up of transferred fragments in the two latest assembly versions (Col and GWH) on chromosome 3 was nearly 0.025%, which was much higher than that of the previously assembled versions (Figure 1c). The number of transferred fragments in *O. sativa* was higher than those in *A. thaliana* (ranging from 1 to 86 in *O. sativa* and 0 to 31 in *A. thaliana*), which appeared to be associated with genome size (~380 Mb for *O. sativa* and ~120 Mb for *A. thaliana*), as has been found in other studies [44,45].

### 3.2. Characterization of the Transfer Fragments and Flanking Sequences

**Figure 2:**
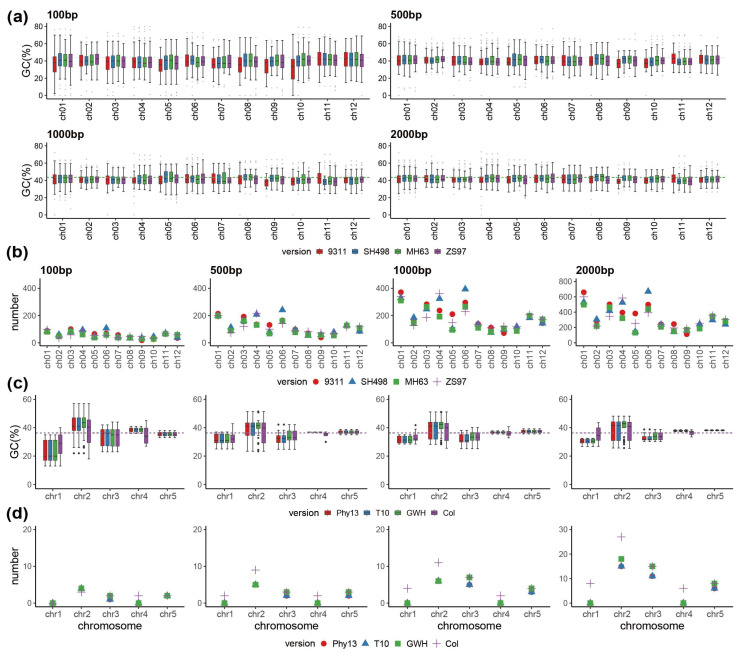
The GC and TE (transposable element) content in flanking sequences around plastome transferred sequences. (**a**,**c**) The GC content of flanking sequences by size category and assembly version in *O. sativa* and *A. thaliana*, respectively. (**b**,**d**) The number of TEs in flanking sequences by size category and assembly version in *O. sativa* and *A. thaliana*, respectively.

In order to further verify whether TEs were associated with transferred sequences, we calculated the GC and TE content in the flanking sequences on both ends of transferred sequences and in the transferred sequences in both *O. sativa* and *A. thaliana* for each insert size category (Figure 2, Supplementary Figure S6). In *O. sativa*, the patterning of GC content in plastome insertions was varied, especially in the case of larger insertions, but not around plastome insertions (Figure 2a, Supplementary Figure S6a). Differences between assembly versions were also more pronounced in *O. sativa* than in *A. thaliana*, both in the case of GC content and TE number. For instance, in version SH498 on chromosome 6, well over 600 TEs were found in the flanking regions for the flanking size category 2000 bp, while in all other versions fewer than 500 were inferred (Figure 2b). The difference in GC content in flanking sequences was similar across insert size categories, but was very different among insertions in *O. sativa* (Figure 2a, Supplementary Figure S6a). The TE content of the transferred sequences fluctuated greatly in the categories of 100–500 bp and 1000 bp+, and the TE content of version ZS97 in chromosome 1 was apparently higher than that of the other three versions in the 1000 bp+ category (Supplementary Figure S6b). The results in *A. thaliana* indicated that GC content flanking plastome insertions in chromosome 2 was elevated more than in any other chromosome, no matter the assembly version or insert size, and the same was true for TE abundance; in addition, the GC content flanking the insertions in chromosome 2 was apparently higher than that within the insertions (Figure 2c,d, Supplementary Figure S6c). The TE number in *A. thaliana* showed a general increase in flanking sequences with recency of assembly version, while GC content was mostly consistent across versions (Figure 2c,d). This result suggested that TEs might be associated with plastome sequence transfer on chromosome 2 in *A. thaliana*. Michalovova et al., verified the correlation between the localization of NUPTs and NUMTs and the distribution of TEs; they showed that the localization of a considerable number of NUPTs and NUMTs was positively correlated with the distribution of TEs in *A. thaliana* and sorghum, and negatively correlated in grape and soybean, and did not correlate in *O. sativa* or maize [46]. Here, we showed the GC content of four assembly versions showed relative fluctuation, and the results of the length and number of TEs in the transferred sequences also differed (Figure 2, Supplementary Figures S6 and S14), indicating that NUPTs were correlated with TE distribution in some instances (such as on chromosome 2 in *A. thaliana*), which is consistent with the previously reported result that the localization of a considerable number of NUPTs correlates with the distribution of TEs [46].

### 3.3. Differences of Plastome to Nuclear Genome IGT among Assembly Versions and Genomic Regions

In order to assess whether IGT from plastome to nucleus showed differences with improvement in the quality of the assembled version of the nuclear genome, we further analyzed the distribution of plastome transfers to the nucleus based on assembly. The results showed that in *A. thaliana*, when the threshold of identity was 95% or higher and the insert length was 100 bp and longer, the frequency of plastome transfer to chromosome 2 was the highest, and that to chromosome 5 was the lowest (Supplementary Figure S7). Among the different *A. thaliana* assembly versions, the transferred fragments with length more than 100 bp and identity more than 95% in the latest no-gap version (Col) on chromosomes 1, 2, and 4 had many more inferred insertions than the older versions (Supplementary Figure S7a,b,d). In *O. sativa*, there were fewer transfers on chromosomes 7 and 8 relative to other chromosomes. The 9311 version and the ZS97 version had large differences in the number of transfers to chromosomes 1 and 10 (Supplementary Figure S8). We further selected several chromosomes and used LINKVIEW v1.0 to visualize the origin and destination of transfers. The results showed that the abundance, location, and frequency of transfers within the same chromosome length range in *A. thaliana* and *O. sativa* were not the same among different assembled versions (Supplementary Figures S9–S11).

**Figure 3:**
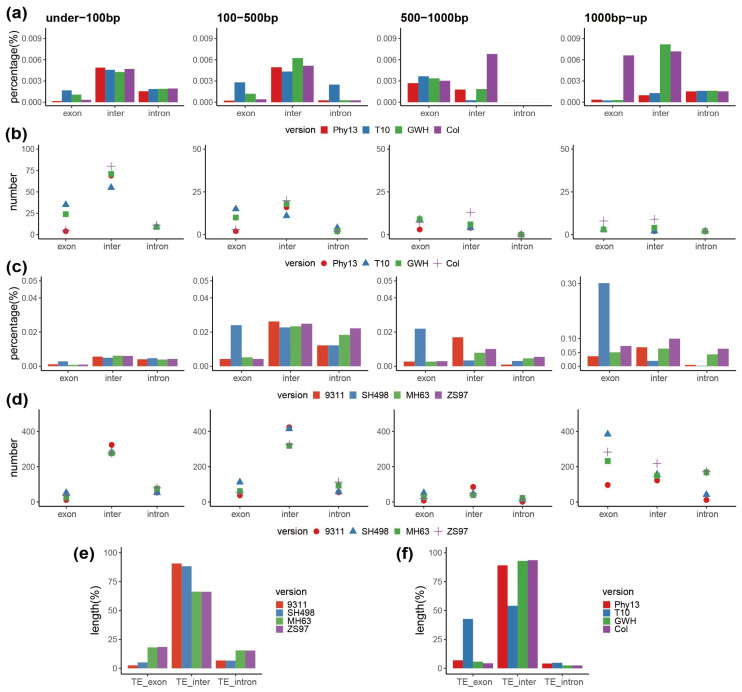
Differences in plastome-to-nuclear genome IGT between different assembly versions in *O. sativa* and *A. thaliana*. (**a**) The length proportion of *A. thaliana* plastome inserts to each genic region in the category with identity ≥ 95%. (**b**) The number of *A. thaliana* plastome inserts to each genic region in the category with identity ≥ 95%. (**c**) The length proportion of *O. sativa* plastome inserts to each genic region in the category with identity ≥ 95%. (**d**) The number of *O. sativa* plastome inserts to each genic region in the category with identity ≥ 95%. Exon refers to the exon region of an annotated gene, intron refers to the intron region of an annotated gene, and inter refers to the intergenic regions between annotated genes. (**e**) TE content of *O. sativa* plastome inserts to each nuclear genic region in the category with identity ≥ 95%. (**f**) TE content of *A. thaliana* plastome inserts to each nuclear genic region in the category with identity ≥ 95%.

To investigate whether the preference of plastome transfer to the nucleus was the same among different versions, we performed intersectional statistics of the relative length and number of plastome sequences transferred to nuclear genomic regions (exon, intron, and intergenic regions) with a sequence identity to the plastome of 95% or higher. The results showed that for the length and number of sequences transferred, the intergenic regions were the most common destination in both *A. thaliana* and *O. sativa*, and there were differences among different assembly versions (Figure 3a–d). In *A. thaliana*, the relative length of transfers in the no-gap version Col was the highest in the 500–1000 bp category, and the least in the 1000 bp+ category in the intergenic regions (Figure 3a), indicating that the length transferred to intergenic regions decreased in the largest fragment category with the improvement of assembly quality, except for the initial version Phy13. In *O. sativa*, the length of the transferred sequences in the 1000 bp+ category accounted for the largest proportion. Except for the original version 9311, the length transferred to the intergenic region showed a relative increase in the four size categories, and the number also increased in the 1000 bp+ category (Figure 3c,d), indicating that the transfer of large fragments within the intergenic regions increases with improvement in the assembly.

We also calculated the ratio of the plastome sequences transferred to the nuclear genomic regions by chromosome. The results showed that most of the plastome transfer was concentrated in the insertion sizes categories of less than 500 bp in *A. thaliana*, and the length of the plastome transfer to chromosome 3 were mainly made up of sequences from the 1000 bp+ category. In *O. sativa*, the lengths of the transferred fragments between chromosomes in each version differed (Supplementary Figure S12a,b). In an effort to find motifs associated with IGT, we also calculated the TE content in the corresponding genomic regions and also the chromosomes mentioned above (Supplementary Figures S12c–h and S13). We found that the TE content of the intergenic regions in both *O. sativa* and *A. thaliana* was higher than that compared to the other two genomic regions (Figure 3e,f; Supplementary Figure S12c,d). In *A. thaliana*, the total length of the plastome sequences transferred to exons in the T10 version were comparatively higher, as was the case with TE content (Figure 3f). In *O. sativa*, the number of transfers to chromosome 6 in the SH498 version was distinctly higher than that in other versions in the 100–500 bp category, and TE content in this category was also obviously higher than that in other versions (Figure 3e), suggesting that TEs were correlated in the same cases with IGT.

## 4. Discussion

### 4.1. IGT Occurs Continuously and Can Be Detected More Accurately with Improved Genome Assembly

IGT from the plastome to the nuclear genome is a dynamic and ongoing process [30,47,48]. Many such transfers have been reported in numerous studies, such as the large and abundant plastome DNA insertions detected in maize nuclear chromosomes [49] and the detection of clustered plastome DNA insertions in *A. thaliana* and *O. sativa* [50]. Our results indicate that plastome DNA transfer is frequent and ongoing, and can be seen in both the original NGS data and the newly published no-gap assembly versions, both in the monocotyledonous *O. sativa* and in the dicotyledonous *A. thaliana*. Such a pattern reveals the importance of organelle-derived fragments as an integral part of the dynamic fragmentation of plant nuclear genomes [26]. With advancements in sequencing technology, the detection of organelle transfer sequences has become more accurate. For instance, the length of a large mitochondrial insertion on nuclear chromosome 2 in *A. thaliana* was initially inferred to be 270 Kb, which was first detected by NGS data, and later found to be 641 Kb using PacBio HiFi sequencing technology [43,51]. We also found that the transfer of plastome to nuclear genome detected in the chromosome 1 of *A. thaliana* was apparently higher in the latest third-generation data Col version than in the NGS data Phy13 version (Figure 1a,b, Supplementary Figure S7a).

### 4.2. TEs May Be a Factor Involved in Mediating IGT

There is no fixed pattern of IGT spanning all plant genomes examined thus far, yet in many cases, certain chromosomes appear to be targeted for insertion. Furthermore, certain genomic regions such as intergenic regions possess more insertions, and many insertions are associated with motifs such as TEs. Furthermore, the effect of the insert appears to differ between larger and smaller insertions in regard to the number of insertions, the proportion of the nuclear chromosome they make up, and the nucleotide composition of flanking sequences. As such, we suspect that differences in transfer abundance is associated with (and possible mediated by) TE content, but this may be simply a correlative pattern wherein certain regions of the genome are inefficient at the removal of both TEs and plastome insertions. From previous reports, the localization of a considerable number of NUPTs and NUMTs was positively correlated with the distribution of TEs in *A. thaliana* and sorghum, and negatively correlated in grape and soybean, implying that recombination around repetitive sequences can lead to rearrangement of chromosomal structure and contribute to various organizational patterns of organelle-derived sequences. Michalovova et al. observed similar distribution patterns of promiscuous DNA in species with different genome sizes and different TE contents, thus suggesting that the distribution pattern of promiscuous DNA does not depend on the abundance or location of TEs in the genome, but reflects the dynamics nature of TE insertion. [46]. Our study shows that the preference for plastome-to-nuclear genome transfer is basically the same (in respect to amplitude, but differs in accuracy) in different assembly versions. For instance, in *A. thaliana*, the plastome-to-nucleus genome transfer has been targeted to chromosome 2, which may be due to the large number of TEs contained in chromosome 2, and transfer occurs mostly in intergenic regions, which may also be mediated by TEs.

### 4.3. IGT as a Possible New Index to Assess Genome Assemblies

Continued organelle sequence transfer and evolution may affect the proper determination of nuclear genes and thus the quality of nuclear genome assembly. As exogenous genetic material to the nuclear genome, the insertion of organellar DNA can lead to host genome instability, and in some cases may lead to structural changes such as recombination of genomic regions, genome size expansion, and heterochromatinization [46,52,53]. The sequence similarity between organelle insertion sequences in the nuclear genome and the original organelle DNA will also decrease with time [45]. Our results suggest that in *A. thaliana* and *O. sativa*, detectable plastome DNA transfer has occurred mostly recently [43,54]. However, it is worth noting that more ancient organellar insertions are more difficult to detect because of mutation and rearrangement obscuring identity [44]. Thus, even in completed nuclear genomes, organelle insertions are unlikely to be fully characterized.

Our research shows that in the iterative process of genome update, plastome DNA transfer can impact nuclear genome assembly and, in turn, the proper inference of plastome transfers, as well as accurate assembly and annotation of the nuclear genome. In addition, the assembly quality can also make the detection of plastid-derived nuclear sequences more accurate. It is hoped that our study demonstrates that inter-version and intra-species comparisons of plastome transfer is an important aspect of accurate genome assembly, as well as being essential for understanding genome evolution. While our study did not compare assembly versions from the same DNA from the same individual plant, and therefore some of the differences between versions are possibly biological in origin, it is clear that some of these differences arose from sequencing and assembly such as seen in the large increase in inferred insertions found on chromosome 1 in *O. sativa* for the 1000 bp+ category in the latest assembly version. Increases in the number of inferred longer insertions is consistent with expectations from long-read sequencing technologies. Additionally, the size and complexity of the genome appears to be associated with differences between versions, as seen between *O. sativa* and *A. thaliana*, which could be from biological and/or assembly sources. Given the findings presented here, we plan to expand the study of plastome insertions to a broader diversity of green plants in an effort to better understand genome coevolution through the recognition of consistent patterns. Such efforts will also help improve assembly algorithms by informing more accurate models about the genomic location, periodicity, and evolution of plastome-to-nuclear genome insertions. With enough baseline data, NUPTs might even be applicable to scoring the completeness of genome assemblies in a manner similar to BUSCO or LAI.

## 5. Conclusions

In this study, we found that IGT from the plastome to the nuclear genome is a dynamic and ongoing process in *O. sativa* and *A. thaliana*, and most detectable transfers have occurred recently. Many differences in inferred transfers between assembly versions likely arose due to differences in sequencing technology and assembly method, but others may be the result of intraspecific variability in insertions. Further work is needed to disentangle biological from assembly-based differences in insertion inference, which is evermore tractable with improvements in sampling, sequencing, and assembly. We also found that NUPTs are often associated with TEs and are more frequently located in intergenic regions. Such findings should be compared across a broader sampling of green plants in an effort to understand how TEs can mediate plastome insertions. Categorizing different insertion types as relates to size, function, and genomic context will be an important step in developing models for their evolution and ultimately genome evolution in general. Any such work is dependent on accurate inference of insertions, and this study provides a starting point for improved determination of insertions into the nuclear genome from the plastome.

## Figures and Tables

**Table 1:** Basic information on *A. thaliana* genomic data.

Version	Name	Time	Ecotype	Assembly	Sequencing Tech	BUSCO
phytozome13	Phy13	2013	Columbia	Athaliana_167.fa.gz	Next-generation sequencing	99.30%
tair10	T10	2014	Columbia	GCA_000001735.2	Next-generation sequencing	99.30%
almost complete	GWH	2021	Columbia	GWHBDNP00000000.1	ONT and PacBio and Hi-C	99.40%
no-gap	Col	2021	Columbia	Col-CEN	ONT and PacBio HiFi long-read	99.40%

**Table 2:** Basic information on *O. sativa* genomic data.

Version	Name	Time	Strain	Assembly	Sequencing Tech	BUSCO
draft sequence	9311	2002	Indica	GCA_000004675.2	Whole-genome shotgun sequencing	96.30%
near complete	SH498	2017	Indica	GCA_002151415.1	PacBio	98.50%
no-gap	MH63	2021	Indica	GCA_001623365.2	PacBio Sequel II	98.70%
no-gap	ZS97	2021	Indica	GCA_001623345.3	PacBio Sequel II	98.70%

## Data Availability

Not applicable.

## Supplementary Materials

The following supporting information can be downloaded at: https://www.mdpi.com/article/10.3390/genes13091620/s1, Figure S1: Results of QUAST assessment of the four versions of the *A. thaliana* nuclear genome; Figure S2: Results of QUAST assessment of the four versions of the *O. sativa* nuclear genome; Figure S3: Collinearity results of pairwise comparison between the four versions of *A. thaliana* nuclear genomes; Figure S4: Collinearity results of pairwise comparison between the four versions of *O. sativa* nuclear genomes; Figure S5: The length ratio and number of transferred plastome sequences in nuclear chromosomes in *O. sativa* and *A. thaliana* with the 80–95% identity; Figure S6: The GC and TE content of the plastome transferred sequences and the flanking sequences around plastome transferred sequences; Figure S7: Distribution of plastome sequences in chromosome 1–5 of four assembled versions of the nuclear genome in *A. thaliana*; Figure S8: Distribution of plastome sequences in chromosomes 01–12 of four assembled versions of the nuclear genome in *O. sativa*; Figure S9: Differences in the distribution of transferred fragments from plastome to chr2 in the same length category of chromosomes in *A. thaliana*; Figure S10: Differences in the distribution of transferred fragments from plastome to chr01 in the same length category of chromosomes in *O. sativa*; Figure S11: Differences in the distribution of transferred fragments from plastome to chr11 in the same length category of chromosomes in *O. sativa*; Figure S12: Correlation analysis of plastome transfer to nuclear genomic regions; Figure S13: The content of TE in the corresponding category of plastome transferred to nuclear chromosomes both in *O. sativa* and *A. thaliana*; Figure S14: Number of TEs on nuclear chromosomes. Table S1: Chromosome length and N counts in the nuclear genomes of *A. thaliana* and *O. sativa*.
