# Supplementary material for: Evaluation of Intracellular Gene Transfers from Plastome to Nuclear Genome across Progressively Improved Assemblies for Arabidopsis thaliana and Oryza sativa
Source: Genes (Basel). 2022 Sep 9;13(9):1620. doi: 10.3390/genes13091620 (PMC9498363; doi:10.3390/genes13091620)
Supplement: Supplementary file 1 [file genes-13-01620-s001.zip › genes-1862854-supplementary/genes-1862854-supplementary(2)/Fig S1.pdf]

Report

	Phy13
# contigs (>= 0 bp)	7
# contigs (>= 1000 bp)	7
# contigs (>= 5000 bp)	7
# contigs (>= 10000 bp)	7
# contigs (>= 25000 bp)	7
# contigs (>= 50000 bp)	7
Total length (>= 0 bp)	119667750
Total length (>= 1000 bp)	119667750
Total length (>= 5000 bp)	119667750
Total length (>= 10000 bp)	119667750
Total length (>= 25000 bp)	119667750
Total length (>= 50000 bp)	119667750
# contigs	7
Largest contig	30427671
Total length	119667750
GC (%)	36.06
N50	23459830
N75	19698289
L50	3
L75	4
# N's per 100 kbp	155.60

All statistics are based on contigs of size >= 500 bp, unless otherwise noted (e.g., "# contigs (>= 0 bp)" and "Total length (>= 0 bp)" include all contigs).

Report

	T10
# contigs (>= 0 bp)	7
# contigs (>= 1000 bp)	7
# contigs (>= 5000 bp)	7
# contigs (>= 10000 bp)	7
# contigs (>= 25000 bp)	7
# contigs (>= 50000 bp)	7
Total length (>= 0 bp)	119668634
Total length (>= 1000 bp)	119668634
Total length (>= 5000 bp)	119668634
Total length (>= 10000 bp)	119668634
Total length (>= 25000 bp)	119668634
Total length (>= 50000 bp)	119668634
# contigs	7
Largest contig	30427671
Total length	119668634
GC (%)	36.06
N50	23459830
N75	19698289
L50	3
L75	4
# N's per 100 kbp	155.60

All statistics are based on contigs of size >= 500 bp, unless otherwise noted (e.g., "# contigs (>= 0 bp)" and "Total length (>= 0 bp)" include all contigs).

Report

	GWH
# contigs (>= 0 bp)	5
# contigs (>= 1000 bp)	5
# contigs (>= 5000 bp)	5
# contigs (>= 10000 bp)	5
# contigs (>= 25000 bp)	5
# contigs (>= 50000 bp)	5
Total length (>= 0 bp)	133725193
Total length (>= 1000 bp)	133725193
Total length (>= 5000 bp)	133725193
Total length (>= 10000 bp)	133725193
Total length (>= 25000 bp)	133725193
Total length (>= 50000 bp)	133725193
# contigs	5
Largest contig	32659241
Total length	133725193
GC (%)	36.34
N50	26161332
N75	22560461
L50	3
L75	4
# N's per 100 kbp	0.15

All statistics are based on contigs of size >= 500 bp, unless otherwise noted (e.g., "# contigs (>= 0 bp)" and "Total length (>= 0 bp)" include all contigs).

Report

	Col
# contigs (>= 0 bp)	7
# contigs (>= 1000 bp)	7
# contigs (>= 5000 bp)	7
# contigs (>= 10000 bp)	7
# contigs (>= 25000 bp)	7
# contigs (>= 50000 bp)	7
Total length (>= 0 bp)	132081078
Total length (>= 1000 bp)	132081078
Total length (>= 5000 bp)	132081078
Total length (>= 10000 bp)	132081078
Total length (>= 25000 bp)	132081078
Total length (>= 50000 bp)	132081078
# contigs	7
Largest contig	32540122
Total length	132081078
GC (%)	36.24
N50	25743512
N75	22217084
L50	3
L75	4
# N's per 100 kbp	0.00

All statistics are based on contigs of size >= 500 bp, unless otherwise noted (e.g., "# contigs (>= 0 bp)" and "Total length (>= 0 bp)" include all contigs).

Figure S1. Results of QUAST assessment of the four versions of the *A. thaliana* nuclear genome.
