# Supplementary material for: Evaluation of Intracellular Gene Transfers from Plastome to Nuclear Genome across Progressively Improved Assemblies for Arabidopsis thaliana and Oryza sativa
Source: Genes (Basel). 2022 Sep 9;13(9):1620. doi: 10.3390/genes13091620 (PMC9498363; doi:10.3390/genes13091620)
Supplement: Supplementary file 1 [file genes-13-01620-s001.zip › genes-1862854-supplementary/genes-1862854-supplementary(2)/Fig S12.pdf]

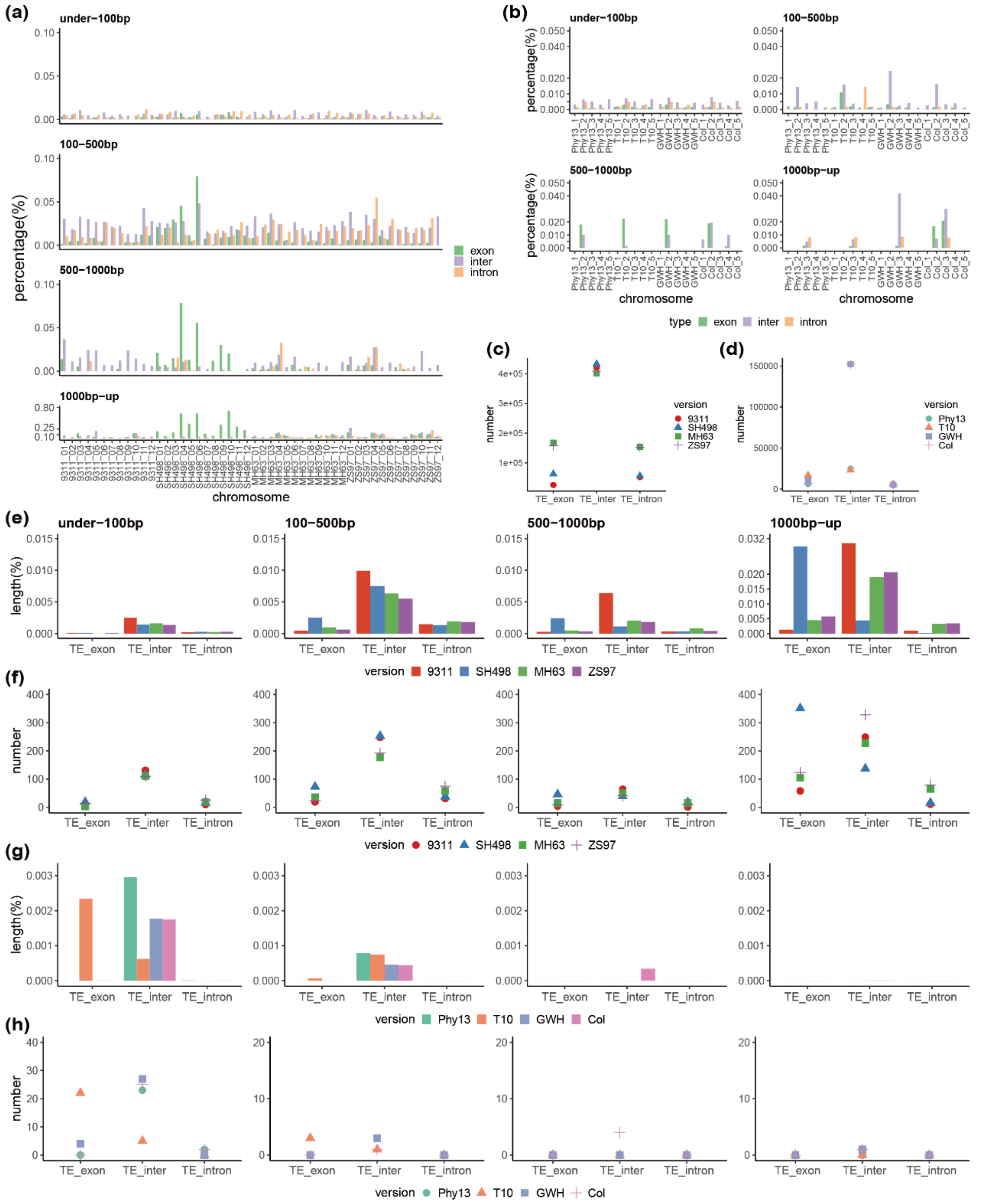

Figure S12. Correlation analysis of plastome transfer to nuclear genomic regions. (a). The length proportion of the plastome transfer to genic regions on each chromosome in *O. sativa*. (b). The length proportion of the plastome transfer to genic regions on each chromosome in *A. thaliana*. (c). The total number of TEs of plastome transferred to genic regions in *O. sativa*. (d). The total number of TEs of plastome transferred to genic regions in *A. thaliana*. (e). Intersection statistics of the length proportion of TEs of plastome transferred to genic regions in *O. sativa.* (f). Intersection statistics of the number of TEs of plastome transferred to genic regions in *O. sativa.* (g). Intersection statistics of the length proportion of TEs of plastome transferred to genic regions of *A. thaliana.* (h). Intersection statistics of the number of TEs of plastome transferred to genic regions of *A. thaliana.*
