# Supplementary material for: Evaluation of Intracellular Gene Transfers from Plastome to Nuclear Genome across Progressively Improved Assemblies for Arabidopsis thaliana and Oryza sativa
Source: Genes (Basel). 2022 Sep 9;13(9):1620. doi: 10.3390/genes13091620 (PMC9498363; doi:10.3390/genes13091620)
Supplement: Supplementary file 1 [file genes-13-01620-s001.zip › genes-1862854-supplementary/genes-1862854-supplementary(2)/Fig S13.pdf]

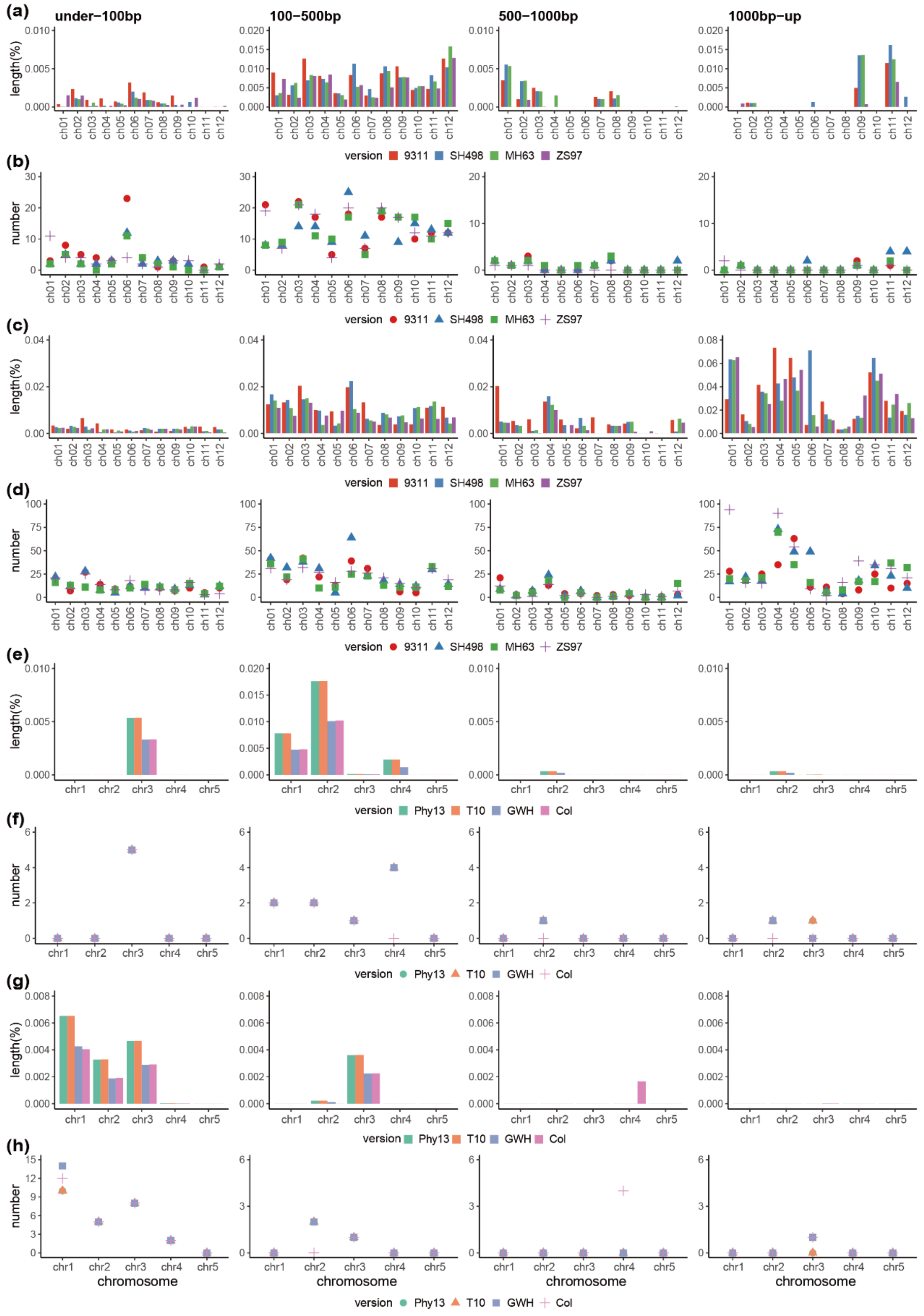

Figure S13. The content of TE in the corresponding category of plastome transferred to nuclear chromosomes both in *O. sativa* and *A. thaliana*. (a). The length proportion of TEs of plastome transferred to nuclear chromosomes in the 80-95% identity category in *O. sativa*. (b). The number of TEs of plastome transferred to nuclear chromosomes in the 80-95% identity category in *O. sativa*. (c). The length proportion of TEs of plastome transferred to nuclear chromosomes in the 95% and

higher identity category in *O. sativa*. (d). The number of TEs of plastome transferred to nuclear chromosomes in the 95% and higher identity category in *O. sativa*. (e). The length proportion of TEs of plastome transferred to nuclear chromosomes in the 80-95% identity category in *A. thaliana*. (f). The number of TEs of plastome transferred to nuclear chromosomes in the 80-95% identity category in *A. thaliana*. (g). The length proportion of TEs of plastome transferred to nuclear chromosomes in the 95% and higher identity category in *A. thaliana*. (h). The number of TEs of plastome transferred to nuclear chromosomes in the 95% and higher identity category in *A. thaliana*.
