# Supplementary material for: Evaluation of Intracellular Gene Transfers from Plastome to Nuclear Genome across Progressively Improved Assemblies for Arabidopsis thaliana and Oryza sativa
Source: Genes (Basel). 2022 Sep 9;13(9):1620. doi: 10.3390/genes13091620 (PMC9498363; doi:10.3390/genes13091620)
Supplement: Supplementary file 1 [file genes-13-01620-s001.zip › genes-1862854-supplementary/genes-1862854-supplementary(2)/Fig S2.pdf]

Report

	9311
# contigs (>= 0 bp)	10490
# contigs (>= 1000 bp)	10490
# contigs (>= 5000 bp)	2745
# contigs (>= 10000 bp)	864
# contigs (>= 25000 bp)	154
# contigs (>= 50000 bp)	37
Total length (>= 0 bp)	426337286
Total length (>= 1000 bp)	426337286
Total length (>= 5000 bp)	402663190
Total length (>= 10000 bp)	390119002
Total length (>= 25000 bp)	379684703
Total length (>= 50000 bp)	375832623
# contigs	10490
Largest contig	47244934
Total length	426337286
GC (%)	43.50
N50	31162561
N75	22947966
L50	6
L75	10
# N's per 100 kbp	3678.09

All statistics are based on contigs of size >= 500 bp, unless otherwise noted (e.g., "# contigs (>= 0 bp)" and "Total length (>= 0 bp)" include all contigs).

Report

	SH498
# contigs (>= 0 bp)	14
# contigs (>= 1000 bp)	14
# contigs (>= 5000 bp)	14
# contigs (>= 10000 bp)	14
# contigs (>= 25000 bp)	14
# contigs (>= 50000 bp)	14
Total length (>= 0 bp)	390983850
Total length (>= 1000 bp)	390983850
Total length (>= 5000 bp)	390983850
Total length (>= 10000 bp)	390983850
Total length (>= 25000 bp)	390983850
Total length (>= 50000 bp)	390983850
# contigs	14
Largest contig	44361539
Total length	390983850
GC (%)	43.62
N50	31778392
N75	29952003
L50	6
L75	9
# N's per 100 kbp	12.79

All statistics are based on contigs of size >= 500 bp, unless otherwise noted (e.g., "# contigs (>= 0 bp)" and "Total length (>= 0 bp)" include all contigs).

Report

	MH63
# contigs (>= 0 bp)	12
# contigs (>= 1000 bp)	12
# contigs (>= 5000 bp)	12
# contigs (>= 10000 bp)	12
# contigs (>= 25000 bp)	12
# contigs (>= 50000 bp)	12
Total length (>= 0 bp)	395765488
Total length (>= 1000 bp)	395765488
Total length (>= 5000 bp)	395765488
Total length (>= 10000 bp)	395765488
Total length (>= 25000 bp)	395765488
Total length (>= 50000 bp)	395765488
# contigs	12
Largest contig	45027022
Total length	395765488
GC (%)	43.65
N50	31921180
N75	30492302
L50	6
L75	9
# N's per 100 kbp	0.00

All statistics are based on contigs of size >= 500 bp, unless otherwise noted (e.g., "# contigs (>= 0 bp)" and "Total length (>= 0 bp)" include all contigs).

Report

	ZS97
# contigs (>= 0 bp)	12
# contigs (>= 1000 bp)	12
# contigs (>= 5000 bp)	12
# contigs (>= 10000 bp)	12
# contigs (>= 25000 bp)	12
# contigs (>= 50000 bp)	12
Total length (>= 0 bp)	391561630
Total length (>= 1000 bp)	391561630
Total length (>= 5000 bp)	391561630
Total length (>= 10000 bp)	391561630
Total length (>= 25000 bp)	391561630
Total length (>= 50000 bp)	391561630
# contigs	12
Largest contig	44754788
Total length	391561630
GC (%)	43.62
N50	32119910
N75	30264782
L50	6
L75	9
# N's per 100 kbp	0.00

All statistics are based on contigs of size >= 500 bp, unless otherwise noted (e.g., "# contigs (>= 0 bp)" and "Total length (>= 0 bp)" include all contigs).

Figure S2. Results of QUAST assessment of the four versions of the *O. sativa* nuclear genome.
