# Supplementary material for: Evaluation of Intracellular Gene Transfers from Plastome to Nuclear Genome across Progressively Improved Assemblies for Arabidopsis thaliana and Oryza sativa
Source: Genes (Basel). 2022 Sep 9;13(9):1620. doi: 10.3390/genes13091620 (PMC9498363; doi:10.3390/genes13091620)
Supplement: Supplementary file 1 [file genes-13-01620-s001.zip › genes-1862854-supplementary/genes-1862854-supplementary(2)/Fig S5.pdf]

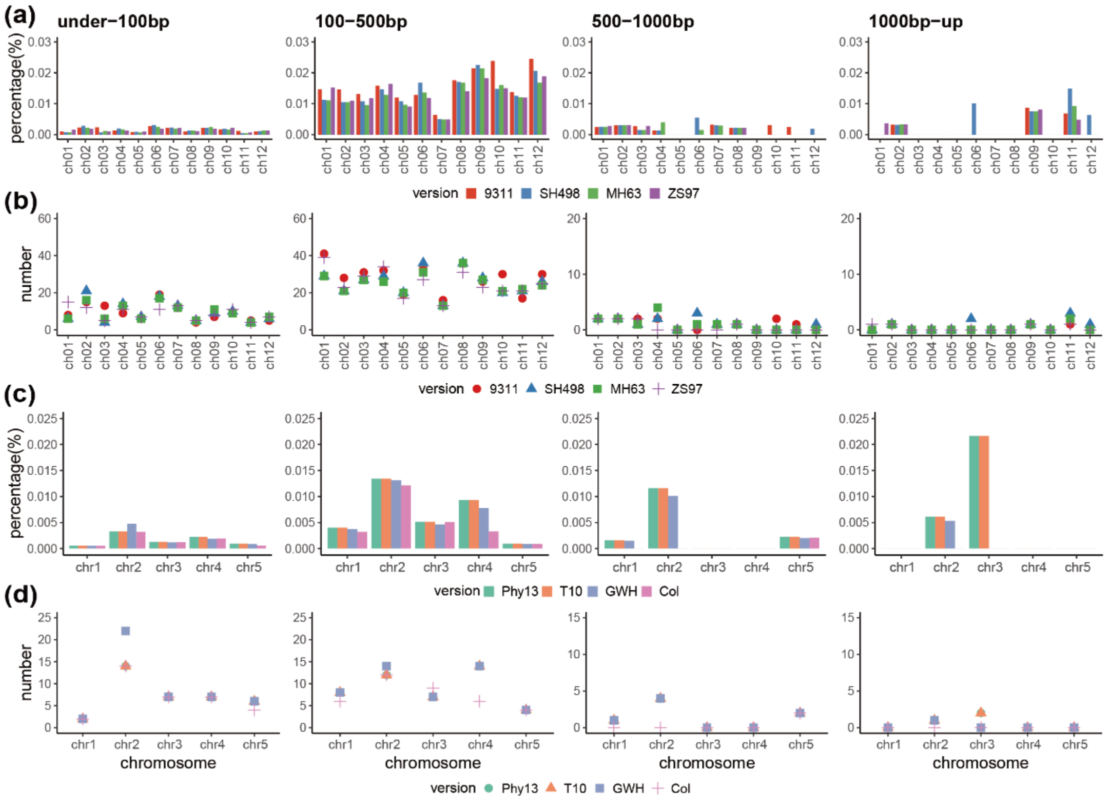

Figure S5. The length ratio and number of transferred plastome sequences in nuclear chromosomes in *O. sativa* and *A. thaliana* with the 80-95% identity. (a). Length proportion of plastome transfers to nuclear chromosomes in *O. sativa* across four different size categories. (b). The number of plastome sequences transferred to nuclear chromosomes in *O. sativa* across four different size categories. (c). Length proportion of plastome transfers to nuclear chromosomes in *A. thaliana* across four different size categories. (d). The number of plastome sequences transferred to nuclear chromosomes in *A. thaliana* across four different size categories. The bar charts represent the relative length of all transferred fragments to the total length of each nuclear chromosome as a percent, assembly versions are ordered along the x axis oldest to newest.
