# Supplementary material for: Evaluation of Intracellular Gene Transfers from Plastome to Nuclear Genome across Progressively Improved Assemblies for Arabidopsis thaliana and Oryza sativa
Source: Genes (Basel). 2022 Sep 9;13(9):1620. doi: 10.3390/genes13091620 (PMC9498363; doi:10.3390/genes13091620)
Supplement: Supplementary file 1 [file genes-13-01620-s001.zip › genes-1862854-supplementary/genes-1862854-supplementary(2)/Fig S6.pdf]

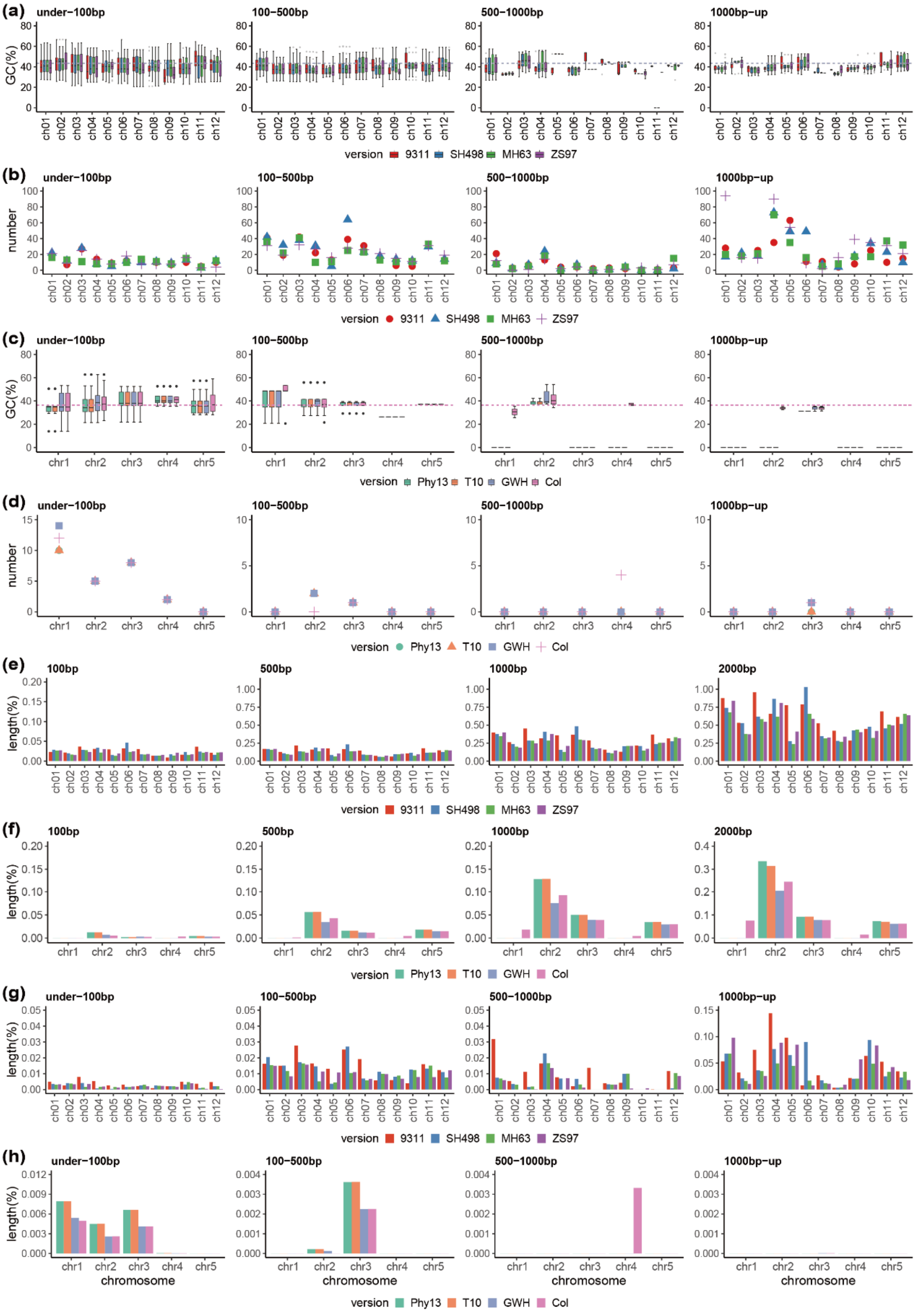

Figure S6. The GC and TE content of the plastome transferred sequences and the flanking sequences around plastome transferred sequences. (a). The GC content of plastome transferred sequences by size category and assembly version in *O. sativa*. (b). The number of TEs of plastome transferred sequences by size category and assembly version in *O. sativa*. (c). The GC content of plastome transferred sequences by size category and assembly version in *A. thaliana*. (d). The

number of TEs of plastome transferred sequences by size category and assembly version in *A. thaliana*. (e). The length proportion of TEs in flanking sequences by size category and assembly version in *O. sativa*. (f). The length proportion of TEs in flanking sequences by size category and assembly version in *A. thaliana*. (g). The length proportion of TEs of plastome transferred sequences by size category and assembly version in *O. sativa*. (h). The length proportion of TEs of plastome transferred sequences by size category and assembly version in *A. thaliana*.
