# Supplementary material for: Evaluation of Intracellular Gene Transfers from Plastome to Nuclear Genome across Progressively Improved Assemblies for Arabidopsis thaliana and Oryza sativa
Source: Genes (Basel). 2022 Sep 9;13(9):1620. doi: 10.3390/genes13091620 (PMC9498363; doi:10.3390/genes13091620)
Supplement: Supplementary file 1 [file genes-13-01620-s001.zip › genes-1862854-supplementary/genes-1862854-supplementary(2)/Fig S7.pdf]

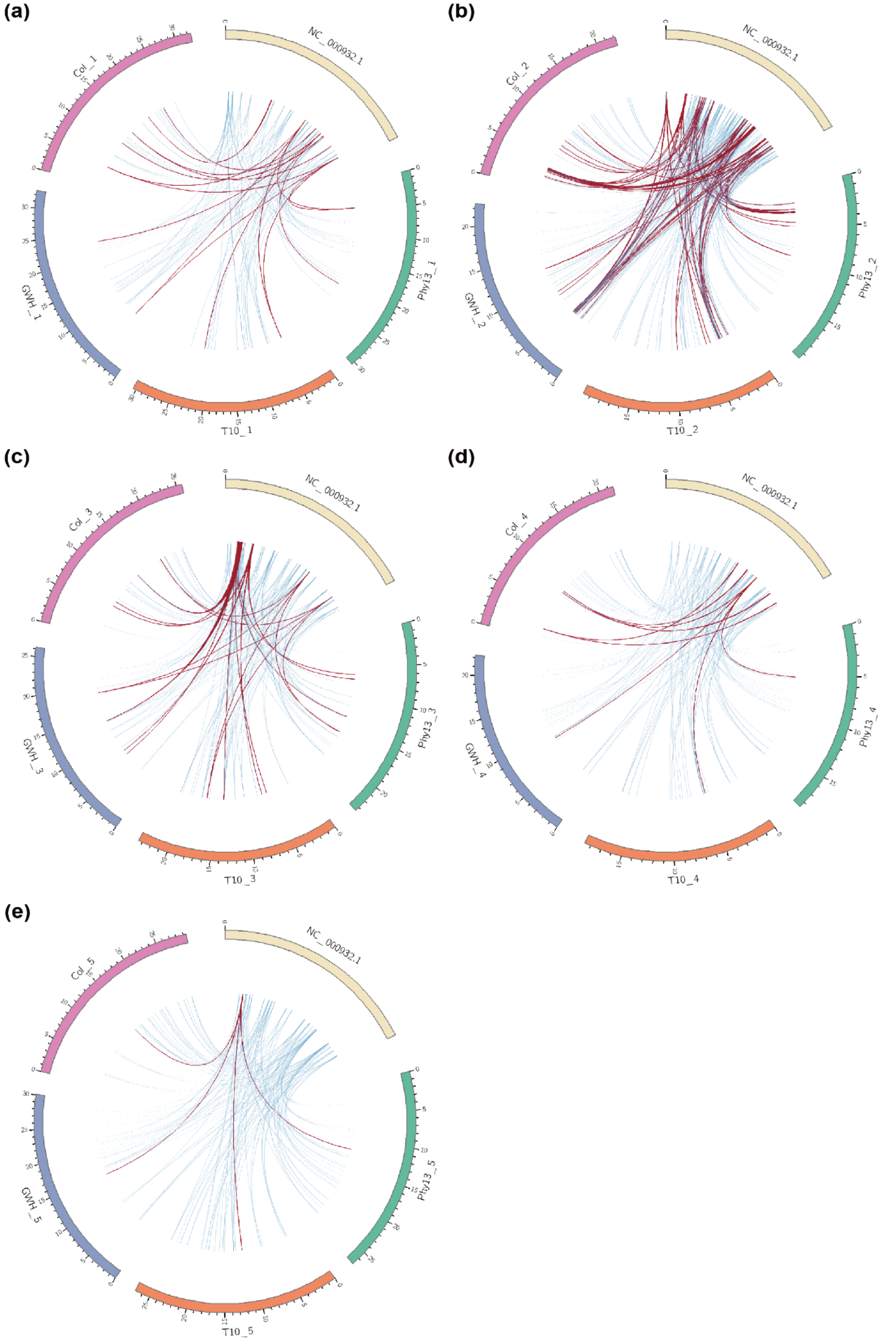

Figure S7. Distribution of plastome sequences in chromosome 1-5 of four assembled versions of the nuclear genome in *A. thaliana*. (a). Distribution of plastome sequences in chromosome 1 of four assembly versions of the nuclear genome in *A. thaliana*. (b). Distribution of plastome sequences in

chromosome 2 of four assembly versions of the nuclear genome in *A. thaliana*. (c). Distribution of plastome sequences in chromosome 3 of four assembly versions of the nuclear genome in *A. thaliana*. (d). Distribution of plastome sequences in chromosome 4 of four assembly versions of the nuclear genome in *A. thaliana*. (e). Distribution of plastome sequences in chromosome 5 of four assembly versions of the nuclear genome in *A. thaliana*. The red lines represent the transferred sequences of plastome-to-chromosome when the identity is greater than 95% and the length is greater than 100 bp, and the blue lines represent all transfer fragments except for the specific category mentioned above when the identity is greater than 80%. Accession number: *A. thaliana*, NC_000932.1.
