# Supplementary material for: Evaluation of Intracellular Gene Transfers from Plastome to Nuclear Genome across Progressively Improved Assemblies for Arabidopsis thaliana and Oryza sativa
Source: Genes (Basel). 2022 Sep 9;13(9):1620. doi: 10.3390/genes13091620 (PMC9498363; doi:10.3390/genes13091620)
Supplement: Supplementary file 1 [file genes-13-01620-s001.zip › genes-1862854-supplementary/genes-1862854-supplementary(2)/Fig S8.pdf]

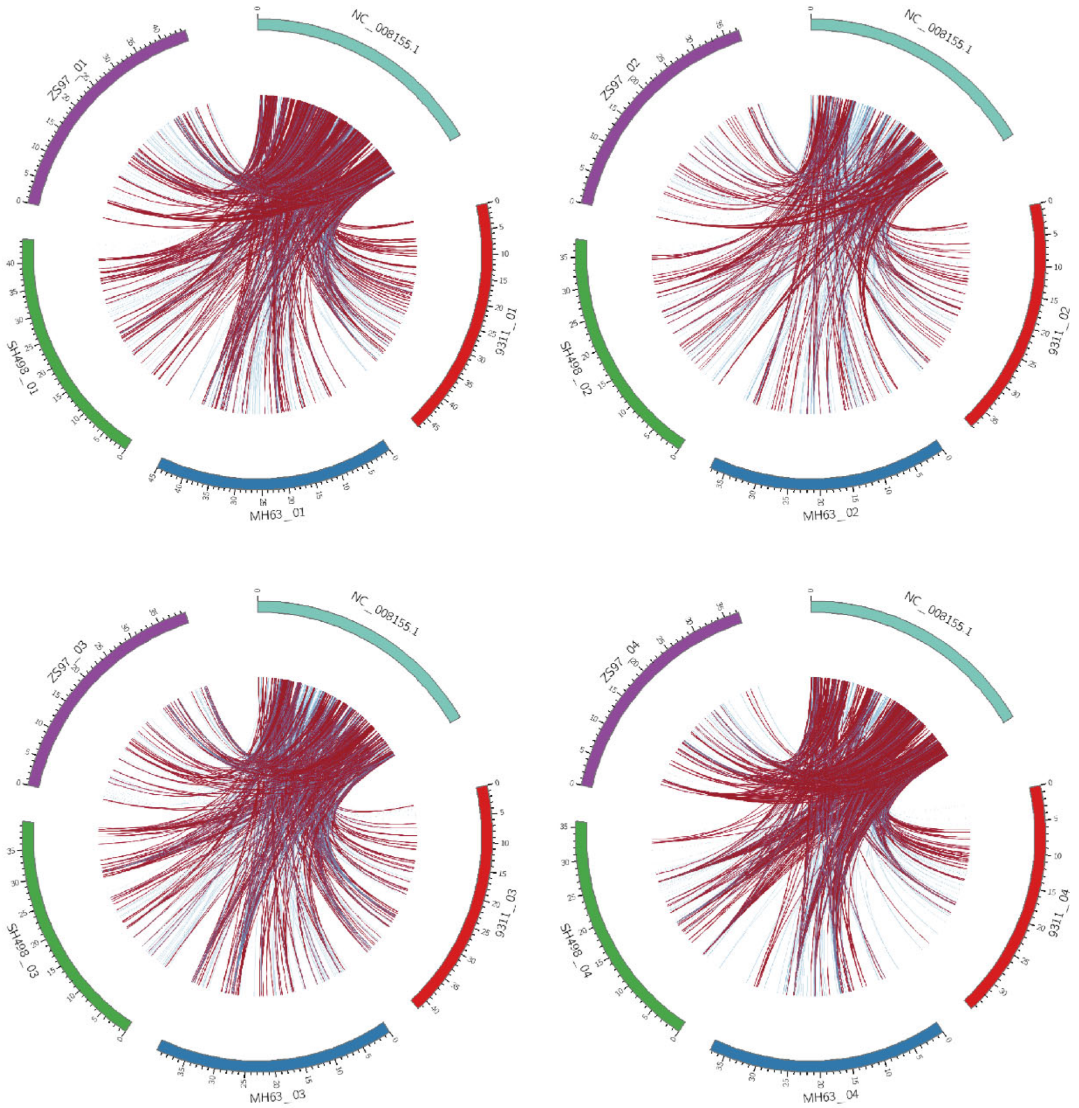
NC_008155.1
ZS97_01
SH498_01
MH63_01
9311_01
NC_008155.1
ZS97_02
SH498_02
MH63_02
9311_02
NC_008155.1
ZS97_03
SH498_03
MH63_03
9311_03
NC_008155.1
ZS97_04
SH498_04
MH63_04
9311_04

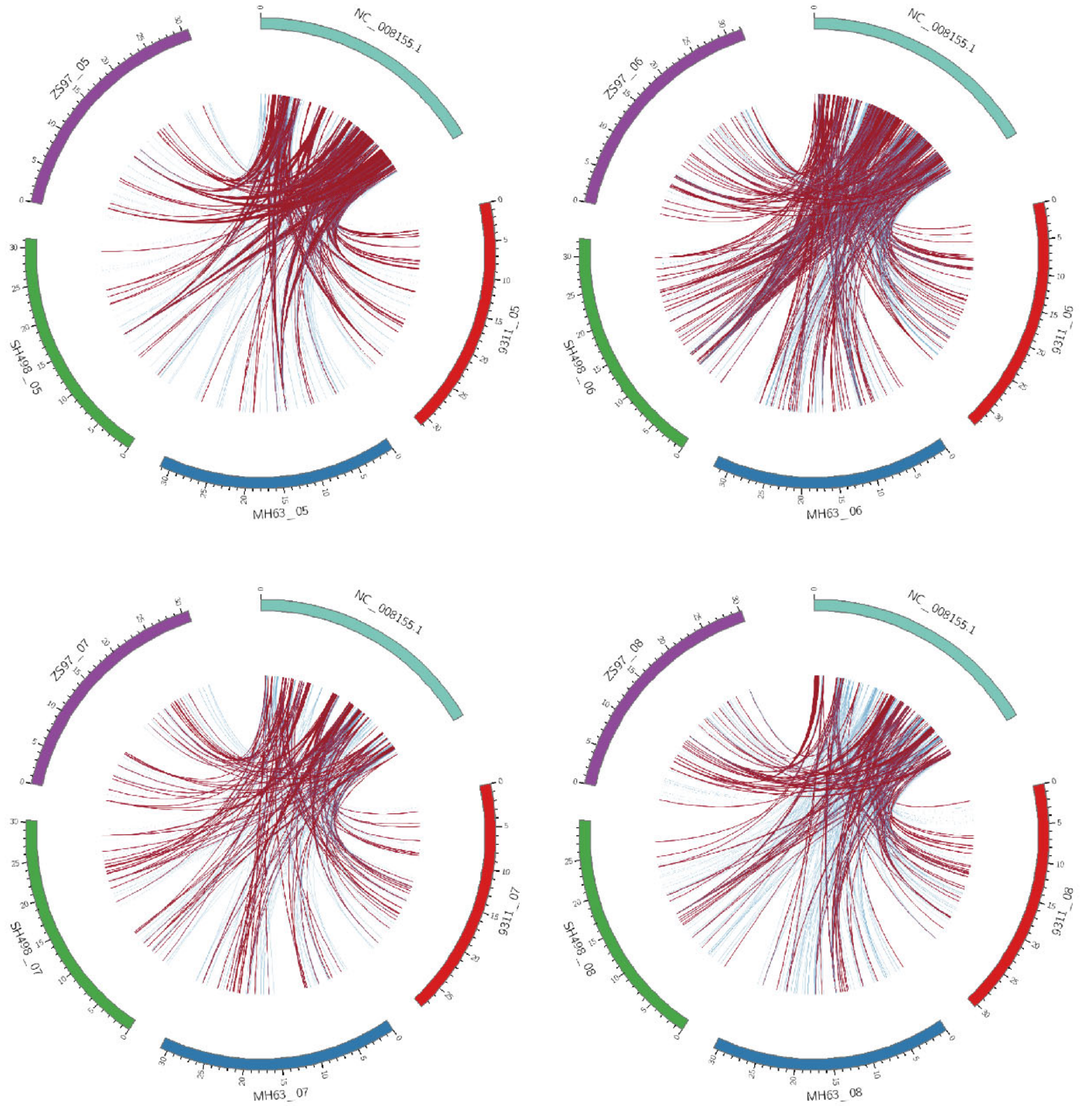
NC_008155.1
ZS97_05
9311_05
SH498_05
MH63_05
NC_008155.1
ZS97_06
9311_06
SH498_06
MH63_06
NC_008155.1
ZS97_07
9311_07
SH498_07
MH63_07
NC_008155.1
ZS97_08
9311_08
SH498_08
MH63_08

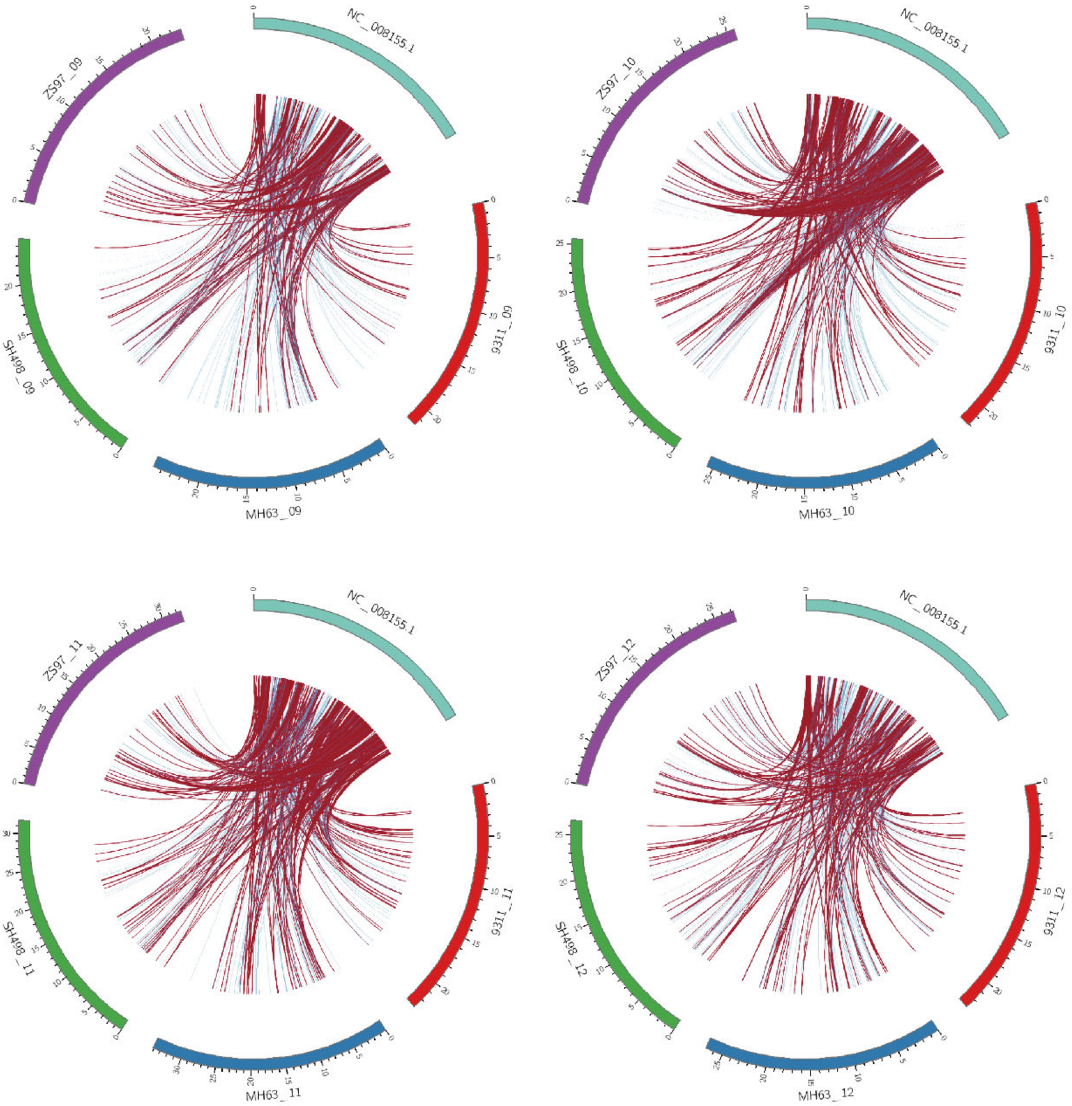

Figure S8. Distribution of plastome sequences in chromosomes 01-12 of four assembled versions of the nuclear genome in *O. sativa*. The red lines represent the transferred sequences of plastome-to-chromosome when the identity is greater than 95% and the length is greater than 100 bp, and the blue lines represent all transfer fragments except for the specific category mentioned above when the identity is greater than 80%. Accession number: *O. sativa*, NC_008155.1.
