# Supplementary material for: Evaluation of Intracellular Gene Transfers from Plastome to Nuclear Genome across Progressively Improved Assemblies for Arabidopsis thaliana and Oryza sativa
Source: Genes (Basel). 2022 Sep 9;13(9):1620. doi: 10.3390/genes13091620 (PMC9498363; doi:10.3390/genes13091620)
Supplement: Supplementary file 1 [file genes-13-01620-s001.zip › genes-1862854-supplementary/genes-1862854-supplementary(2)/Fig S9.pdf]

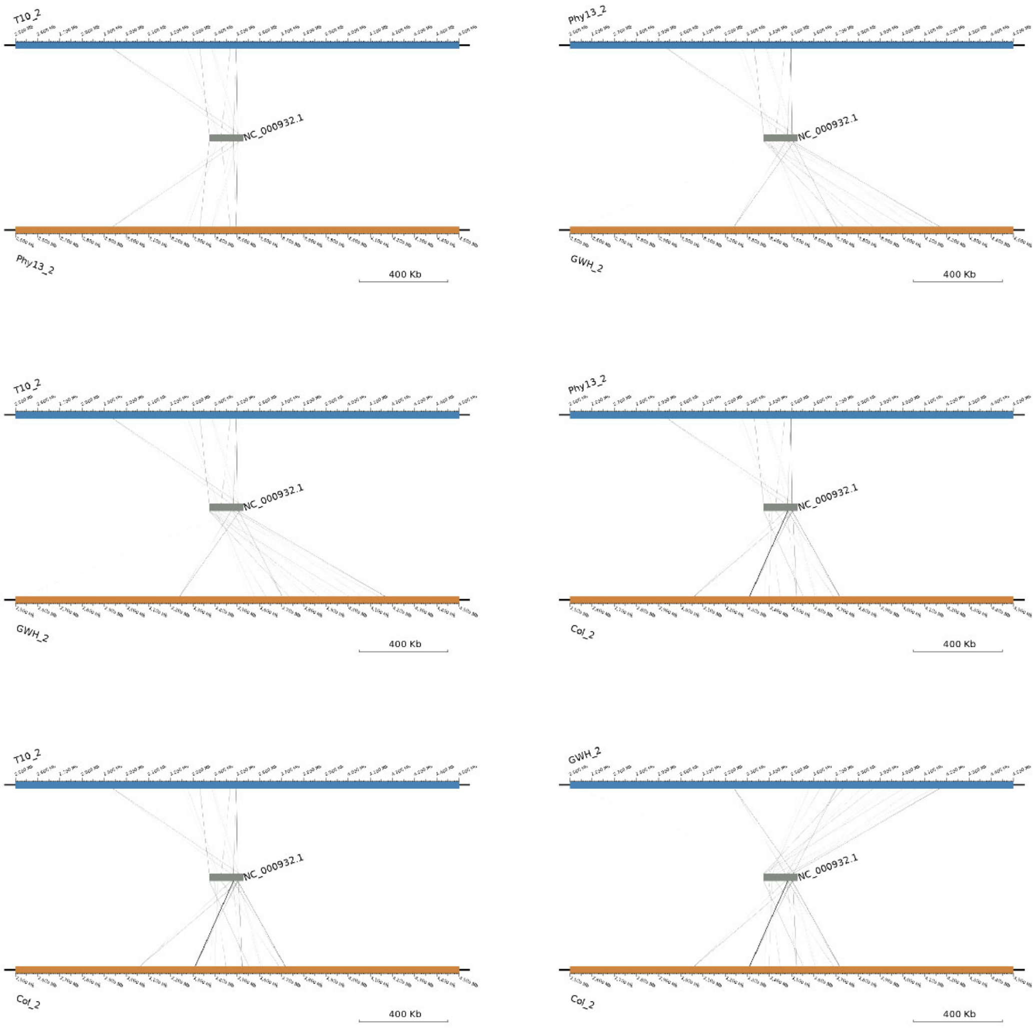

Figure S9. Differences in the distribution of transferred fragments from plastome to chr2 in the same length category of chromosomes in *A. thaliana*.
